# Supplementary material for: A Benchmark for Virus Infection Reporter Virtual Staining in Fluorescence and Brightfield Microscopy
Source: Sci Data. 2025 May 28;12:886. doi: 10.1038/s41597-025-05194-3 (PMC12120016; doi:10.1038/s41597-025-05194-3)
Supplement: Supplementary file 1 — Supplementary information to the manuscript: A Benchmark for Virus Infection Reporter Virtual Staining in Fluorescence and Brightfield Microscopy [file 41597_2025_5194_MOESM1_ESM.pdf]

Supplementary information to the manuscript: A Benchmark for Virus Infection Reporter Virtual Staining in Fluorescence and Brightfield Microscopy

Authors: Maria Wyrzykowska*, Gabriel della Maggiora*, Nikita Deshpande, Ashkan Mokarian, Artur Yakimovich
*these authors contributed equally to this work

correspondence: a.yakimovich@hzdr.de

Standard Deviation Across Dataset Splits

To evaluate the impact of data splitting on model performance, we trained and tested the U-Net model for 100000 steps on datasets' splits generated using three different random seeds (43, 44, and 45) for each virus. For viruses represented as time-lapse sequences, we ensured that the same sample across different timepoints was included in only one of the data splits to prevent data leakage.

We then computed the mean and standard deviation of the performance metrics across all models and datasets to assess variability in model performance. The results are summarised in Table S1. The analysis indicates that while certain datasets, notably VACV and HAdV, exhibit higher variability across different data splits, particularly concerning the PSNR metric. Such fluctuations are generally limited. This consistency across most metrics and datasets reinforces the robustness and reliability of the study's conclusions.

Table S1. Performance of the U-Net model trained on HAdV, HAdV (2ch), VACV, HSV, IAV, and RV datasets. Reported metrics include the mean and standard deviation across models trained with three different data split seeds. Metrics shown are MSE, PSNR, and SSIM, along with their corresponding foreground (FG) and background (BG) versions.

Dataset	MSE (↓)	PSNR (↑)	SSIM (↑)	BG MSE (↓)	BG PSNR (↑)	BG SSIM (↑)	FG MSE (↓)	FG PSNR (↑)	FG SSIM (↑)
HAdV	0.029 ± 0.002	25.282 ± 0.343	0.897 ± 0.007	0.006 ± 0.002	29.240 ± 1.262	0.952 ± 0.003	0.102 ± 0.006	16.906 ± 0.071	0.724 ± 0.010
HAdV (2ch)	0.032 ± 0.004	24.865 ± 1.078	0.892 ± 0.013	0.008 ± 0.005	28.210 ± 2.765	0.948 ± 0.008	0.105 ± 0.002	16.635 ± 0.260	0.714 ± 0.022
VACV	0.046 ± 0.005	23.963 ± 1.539	0.861 ± 0.013	0.012 ± 0.005	27.323 ± 1.863	0.970 ± 0.010	0.156 ± 0.022	14.379 ± 0.685	0.492 ± 0.044
HSV	0.011 ± 0.000	29.465 ± 0.350	0.935 ± 0.002	0.001 ± 0.000	35.885 ± 0.114	0.974 ± 0.001	0.096 ± 0.001	16.555 ± 0.017	0.540 ± 0.004
IAV	0.040 ± 0.002	24.609 ± 0.160	0.907 ± 0.002	0.001 ± 0.000	35.950 ± 0.713	0.974 ± 0.000	0.521 ± 0.025	9.267 ± 0.178	0.216 ± 0.011
RV	0.025 ± 0.001	24.681 ± 0.163	0.913 ± 0.002	0.003 ± 0.001	31.946 ± 0.989	0.958 ± 0.002	0.419 ± 0.014	9.862 ± 0.157	0.205 ± 0.004

Background-foreground metrics

To gain a more comprehensive understanding of the models' behavior, we employed additional sets of metrics. We calculated traditional metrics on the data segmented into "foreground" and "background" using a threshold-based approach. Specifically, regions of an image with values higher than a certain manually chosen threshold in the ground truth viral signal channel were classified as "foreground," while the rest were classified as "background." The threshold was set to -0.8 for all viruses except HAdV, for which we used -0.9. Table S2 contains the results.

The results suggest that predicting the background is easier, as shown by lower MSE and higher PSNR and SSIM scores across all models. VACV predictions had notably higher MSE, likely due to overestimating infection extent, which was confirmed by a manual review. Foreground metrics were worse overall, with HSV and HAdV performing best, while IAV and RV performed the worst, likely due to models hallucinating the signal. U-Net generally outperformed pix2pix, except for IAV, where pix2pix produced clearer, more accurate results despite U-Net's blurrier predictions.

Cell classification metrics

Table S3 reports IoU, F1, accuracy, precision and recall metrics, but based on whole-cell masks, as opposed to previously used nuclei masks, which were available only for the HAdV and HAdV (2ch) datasets. The conclusions remain consistent: pix2pix generally outperforms U-Net, except in accuracy for both datasets and precision for the HAdV (2ch) data. Pix2pix achieves better IoU, F1, and recall scores, with precision being either comparable or slightly lower for HAdV. The accuracy, however, is lower when compared to metrics calculated from the nuclei masks. This can be attributed to the larger size of cells, which makes it easier to achieve higher IoU and recall scores. Because cells occupy a larger portion of the image, achieving high accuracy, which is heavily influenced by the background, becomes more challenging.

Table S2. Performance of U-Net and pix2pix models, trained on HAdV, HAdV (2ch), VACV, HSV, IAV and RV data. Metrics reported are MSE, PSNR and SSIM, calculated separately for background (BG) or foreground (FG). For pix2pix, we report the mean and standard deviation of each metric based on the 3 trials.

Dataset	Model	BG MSE (↓)	BG PSNR (↑)	BG SSIM (↑)	FG MSE (↓)	FG PSNR (↑)	FG SSIM (↑)
HAdV	pix2pix	0.012 ± 4.0e-05	26.272 ± 3.8e-02	0.923 ± 3.0e-05	0.092 ± 2.0e-04	17.044 ± 2.0e-02	0.686 ± 1.0e-04
	U-Net	**0.007**	**28.435**	**0.950**	**0.087**	**17.500**	**0.738**
HAdV (2ch)	pix2pix	0.010 ± 3.0e-05	**27.597 ± 3.3e-02**	0.944 ± 6.0e-05	0.097 ± 8.0e-04	16.882 ± 1.7e-02	0.716 ± 1.0e-03
	U-Net	**0.009**	27.263	**0.947**	**0.094**	**16.989**	**0.726**
VACV	pix2pix	0.057 ± 2.0e-05	21.144 ± 5.0e-04	0.919 ± 5.0e-05	0.201 ± 6.0e-04	13.304 ± 1.4e-02	0.426 ± 1.6e-03
	U-Net	**0.031**	**26.673**	**0.949**	**0.163**	**14.054**	**0.520**
HSV	pix2pix	0.003 ± 2.0e-06	32.252 ± 7.0e-03	0.961 ± 1.0e-05	0.143 ± 8.0e-06	14.607 ± 5.0e-04	0.438 ± 6.0e-05
	U-Net	**0.002**	**35.137**	**0.974**	**0.089**	**16.823**	**0.541**
IAV	pix2pix	0.019 ± 2.0e-05	23.403 ± 6.0e-03	0.942 ± 3.0e-05	**0.498 ± 2.0e-04**	**9.429 ± 2.0e-03**	0.174 ± 8.0e-06
	U-Net	**0.001**	**35.856**	**0.974**	0.544	9.075	**0.208**
RV	pix2pix	0.022 ± 9.0e-06	22.622 ± 2.0e-03	0.903 ± 2.0e-05	0.465 ± 1.0e-04	9.377 ± 9.6e-04	0.160 ± 1.4e-04
	U-Net	**0.002**	**33.174**	**0.960**	**0.438**	**9.691**	**0.205**

Table S3. Performance of U-Net and pix2pix models, trained on HAdV and HAdV (2ch) data. Metrics reported are IoU, F1, accuracy, precision, and recall, calculated based on cells segmentation masks. For pix2pix, we report the mean and standard deviation of each metric based on 3 trials.

Dataset	Model	IoU (↑)	F1 (↑)	Accuracy (↑)	Precision (↑)	Recall (↑)
HAdV	pix2pix	**0.326 ± 6.0e-04**	**0.462 ± 6.0e-04**	0.856 ± 2.0e-04	0.500 ± 7.0e-04	**0.548 ± 6.0e-04**
	U-Net	0.303	0.440	**0.871**	**0.528**	0.447
HAdV (2ch)	pix2pix	**0.294 ± 2.0e-03**	**0.428 ± 2.0e-03**	0.868 ± 4.0e-04	**0.529 ± 3.0e-03**	**0.438 ± 2.0e-03**
	U-Net	0.282	0.413	**0.871**	0.516	0.408

Standard Deviation Across Samples

We calculated the standard deviations of performance metrics across all models and datasets to assess the variation in model performance. The results are detailed in Tables S4 and S5.

The analysis reveals that VACV exhibits the highest variance in both general and background metrics. This suggests that the models made substantial errors on a few samples, likely exacerbated by the smaller size of the VACV test set. In contrast, the standard deviations for background metrics across other viruses were relatively low. However, foreground metrics showed higher variability, with IAV demonstrating the greatest standard deviation in MSE.

Table S4. Performance of U-Net and pix2pix models, trained on HAdV, HAdV (2ch), VACV, HSV, IAV, and RV data. Metrics reported are the mean and standard deviation between results for each datapoint for MSE, PSNR, and SSIM, along with their foreground (FG) and background (BG) versions.

Dataset	Model	MSE (↓)	PSNR (↑)	SSIM (↑)	BG MSE (↓)	BG PSNR (↑)	BG SSIM (↑)	FG MSE (↓)	FG PSNR (↑)	FG SSIM (↑)
HAdV	pix2pix	0.034 ± 0.032	24.214 ± 4.218	0.870 ± 0.060	0.012 ± 0.009	26.272 ± 3.267	0.923 ± 0.016	0.092 ± 0.055	17.044 ± 2.433	0.686 ± 0.084
	U-Net	**0.031 ± 0.038**	**25.331 ± 4.753**	**0.899 ± 0.067**	**0.007 ± 0.006**	**28.435 ± 3.112**	**0.950 ± 0.011**	**0.087 ± 0.060**	**17.500 ± 2.796**	**0.738 ± 0.076**
HAdV (2ch)	pix2pix	**0.032 ± 0.032**	**24.824 ± 4.776**	0.892 ± 0.065	0.010 ± 0.007	**27.597 ± 3.954**	0.944 ± 0.012	0.097 ± 0.057	16.882 ± 2.698	0.716 ± 0.083
	U-Net	**0.032 ± 0.034**	24.767 ± 4.485	**0.895 ± 0.067**	**0.009 ± 0.006**	27.263 ± 3.056	**0.947 ± 0.009**	**0.094 ± 0.057**	**16.989 ± 2.542**	**0.726 ± 0.078**
VACV	pix2pix	0.083 ± 0.070	21.087 ± 3.593	0.825 ± 0.096	0.057 ± 0.069	21.144 ± 4.480	0.919 ± 0.073	0.201 ± 0.078	13.304 ± 1.621	0.426 ± 0.049
	U-Net	**0.057 ± 0.047**	**24.563 ± 7.304**	**0.860 ± 0.090**	**0.031 ± 0.048**	**26.673 ± 6.911**	**0.949 ± 0.059**	**0.163 ± 0.044**	**14.054 ± 1.195**	**0.520 ± 0.068**
HSV	pix2pix	0.016 ± 0.007	27.514 ± 2.647	0.917 ± 0.030	0.003 ± 0.002	32.252 ± 2.108	0.961 ± 0.015	0.143 ± 0.037	14.607 ± 1.055	0.438 ± 0.062
	U-Net	**0.010 ± 0.005**	**29.673 ± 2.714**	**0.938 ± 0.023**	**0.002 ± 0.002**	**35.137 ± 2.704**	**0.974 ± 0.010**	**0.089 ± 0.034**	**16.823 ± 1.503**	**0.541 ± 0.073**
IAV	pix2pix	0.054 ± 0.014	22.266 ± 1.103	0.875 ± 0.023	0.019 ± 0.004	23.403 ± 0.855	0.942 ± 0.007	**0.498 ± 0.211**	**9.429 ± 1.818**	0.174 ± 0.088
	U-Net	**0.041 ± 0.016**	**24.374 ± 1.861**	**0.905 ± 0.023**	**0.001 ± 0.000**	**35.856 ± 1.587**	**0.974 ± 0.003**	0.544 ± 0.235	9.075 ± 1.887	**0.208 ± 0.109**
RV	pix2pix	0.045 ± 0.009	21.668 ± 0.927	0.863 ± 0.019	0.022 ± 0.004	22.622 ± 0.818	0.903 ± 0.011	0.465 ± 0.056	9.377 ± 0.546	0.160 ± 0.028
	U-Net	**0.024 ± 0.007**	**24.738 ± 1.400**	**0.917 ± 0.018**	**0.002 ± 0.001**	**33.174 ± 1.650**	**0.960 ± 0.007**	**0.438 ± 0.088**	**9.691 ± 0.888**	**0.205 ± 0.053**

Table S5. Performance of U-Net and pix2pix models, trained on HAdV, HAdV (2ch), HSV, IAV, and RV data. Metrics reported are the mean and standard deviation between results for each datapoint for IoU, F1, accuracy, precision, and recall calculated based on the nuclei masks.

Dataset	Model	IoU (↑)	F1 (↑)	Accuracy (↑)	Precision (↑)	Recall (↑)
HAdV	pix2pix	**0.300 ± 0.163**	**0.437 ± 0.199**	0.959 ± 0.022	0.503 ± 0.302	**0.473 ± 0.141**
	U-Net	0.268 ± 0.137	0.404 ± 0.174	0.963 ± 0.025	**0.557 ± 0.309**	0.357 ± 0.124
HAdV (2ch)	pix2pix	**0.239 ± 0.138**	**0.364 ± 0.182**	0.960 ± 0.025	**0.524 ± 0.320**	**0.322 ± 0.141**
	U-Net	0.235 ± 0.140	0.360 ± 0.183	**0.961 ± 0.026**	0.522 ± 0.321	0.306 ± 0.129
HSV	pix2pix	0.660 ± 0.054	0.794 ± 0.040	0.985 ± 0.005	**0.862 ± 0.052**	0.741 ± 0.063
	U-Net	**0.727 ± 0.048**	**0.841 ± 0.034**	**0.988 ± 0.005**	0.858 ± 0.050	**0.830 ± 0.056**
IAV	pix2pix	**0.249 ± 0.082**	**0.391 ± 0.107**	0.967 ± 0.008	0.485 ± 0.154	**0.341 ± 0.095**
	U-Net	0.174 ± 0.067	0.290 ± 0.098	**0.971 ± 0.010**	**0.655 ± 0.186**	0.190 ± 0.072
RV	pix2pix	0.219 ± 0.047	0.357 ± 0.062	0.963 ± 0.015	0.347 ± 0.088	**0.379 ± 0.056**
	U-Net	**0.294 ± 0.085**	**0.448 ± 0.104**	**0.978 ± 0.008**	**0.614 ± 0.083**	0.358 ± 0.102
